# Recent advances in T-cell engineering for use in immunotherapy

**DOI:** 10.12688/f1000research.9073.1

**Published:** 2016-09-19

**Authors:** Preeti Sharma, David M. Kranz

**Affiliations:** 1 Department of Biochemistry, University of Illinois, Urbana, IL, USA

**Keywords:** immunotherapy, T cell, receptor, neoantigens, chimeric antigen receptor

## Abstract

Adoptive T-cell therapies have shown exceptional promise in the treatment of cancer, especially B-cell malignancies. Two distinct strategies have been used to redirect the activity of *ex vivo *engineered T cells. In one case, the well-known ability of the T-cell receptor (TCR) to recognize a specific peptide bound to a major histocompatibility complex molecule has been exploited by introducing a TCR against a cancer-associated peptide/human leukocyte antigen complex. In the other strategy, synthetic constructs called chimeric antigen receptors (CARs) that contain antibody variable domains (single-chain fragments variable) and signaling domains have been introduced into T cells. Whereas many reviews have described these two approaches, this review focuses on a few recent advances of significant interest. The early success of CARs has been followed by questions about optimal configurations of these synthetic constructs, especially for efficacy against solid tumors. Among the many features that are important, the dimensions and stoichiometries of CAR/antigen complexes at the synapse have recently begun to be appreciated. In TCR-mediated approaches, recent evidence that mutated peptides (neoantigens) serve as targets for endogenous T-cell responses suggests that these neoantigens may also provide new opportunities for adoptive T-cell therapies with TCRs.

## Introduction

T cells recognize antigens as short peptides bound to a protein encoded by the major histocompatibility complex (MHC). The key T-cell molecule involved in binding to the peptide-MHC (pepMHC) is an αβ heterodimer called the T-cell receptor (TCR) ^1^. The TCR is part of a cell surface complex with subunits of CD3, which provide the proximal signaling components. In addition, co-receptors CD4 and CD8 are involved in the recognition of class II and class I MHC, respectively. TCRs have evolved to recognize MHC molecules such that during thymic development, only T cells with TCRs that bind to MHC will be exported to the periphery as mature T cells (termed “positive” selection) ^2^. T cells with TCRs that bind self-peptide/MHC with too high affinity will be deleted in the thymus (termed “negative” selection). The TCR affinities that trigger signaling in these processes are very low, and the range that distinguishes positive selection from negative selection or peripheral T-cell activation appears to be relatively narrow and most likely influenced by the strength of the peptide:MHC interaction ^3– 5^.

The remarkable feature of the system is that despite their low affinities, TCRs mediate peptide-specific reactivity with very low levels of the pepMHC on the antigen-presenting cells (APCs). Thus, a T cell can be triggered with a TCR affinity of 100 μM and only a few pepMHC complexes on the surface of the target cell ^5– 9^. This exquisite sensitivity allows the recognition of virtually any intracellular foreign peptide that can be bound to an MHC molecule and transported to the surface. Given these properties, the use of TCRs that recognize tumor-antigen peptides has become an important strategy for adoptive T-cell therapies. Clinical efforts to date have focused on shared self-peptides that are from proteins upregulated in some cancers, such as WT1 antigen, differentiation antigens like gp100 and MART-1, and cancer/testis antigens like NY-ESO and MAGE-A3 (for example, ^10– 16^). However, a significant challenge in adoptive T-cell therapy with gene-transferred TCRs is competition for pairing with the endogenous TCR chains, leading to lower levels of the tumor-specific TCR or possibly off-target reactivities of mispaired TCRs that lead to graft-versus-host reactions ^17^. Current strategies to minimize or avoid mispairing include the use of cysteines in exogenous TCR constant domains that promote preferential pairing ^18– 20^ or gene editing strategies that limit the expression of the endogenous TCR chains ^21, 22^.

The other strategy for redirecting the activity of T cells has been to create synthetic receptors that use an antibody fragment (single-chain fragments variable, or scFv) fused to a transmembrane region and signaling domains ^23^. These chimeric antigen receptors (CARs) have allowed T cells to be targeted against cancers in an MHC-independent mechanism. The most clinically studied CARs are those that target B-cell malignancies, in particular CARs against the antigen CD19 (for example, ^24– 30^). However, there is intense interest in developing CARs with other specificities, especially those against cell surface antigens expressed on solid tumors (for example, ErbB2, mesothelin, EGFR, and Tn-glycopeptides ^31– 36^).

Given the clinical progress with TCR- and CAR-mediated therapies, it is not surprising that there have been numerous reviews on these adoptive T-cell approaches (for example, ^37– 42^). Reviews have covered many of the properties that might guide the optimal configuration and application of these receptors, including binding affinities, specificity, construct design, signaling domains (CARs), vector delivery systems, recipient T-cell populations, and manufacturing. Arguably, one of the most important elements of each adoptive T-cell therapy strategy is the choice of target, whether it is a pepMHC for TCR therapies or a cell surface antigen for CAR therapies. With this in mind, we focus here on three features of TCR- or CAR-mediated therapies that have received recent attention: (1) dimensions of the TCR/pepMHC versus CAR/antigen complexes at the synapse, (2) target antigen sensitivity and affinity requirements of T cells expressing TCRs and CARs, and (3) mutated cancer peptides (neoantigens) as targets for adoptive T-cell therapies with TCRs.

## Dimensions of TCR versus CAR interfaces

Whereas the TCR/CD3 complex has evolved to be an exquisitely sensitive recognition and signaling machine, CARs represent synthetic constructs with distinct differences: antigen binding is accomplished with an scFv, membrane insertion is accomplished using the membrane-spanning region of yet another T-cell molecule (for example, CD8), and intracellular signaling components are derived from multiple T-cell molecules, often the CD3ζ subunit and CD28, 4-1BB, or OX40. This structural organization endows CARs with MHC-independent binding properties of antibodies and unique signaling properties that share some of the features of normal T cells but that differ in both quantitative and mechanistic details (for example, ^37– 42^).

Although the TCR/CD3 complex fixes the mechanisms involved in TCR-mediated adoptive T-cell approaches, including the maximal surface level of the complex ^43^, the design of CARs and their inherent mechanisms continue to evolve. First-generation CARs consisted of an scFv linked to the CD3ζ subunit in order to more closely mimic the natural TCR/CD3 signaling machinery, in principle allowing clustering and cross-linking of multiple receptors in the membrane. Improvements in signaling capacity and T-cell function were achieved in second- and third-generation CARs by adding the signaling domain(s) of CD28, 4-1BB, or OX40 or a combination of these. These domains were designed to incorporate co-stimulatory signaling (signal 2). Hinge/spacer domains in CARs enable stable expression and typically consist of a hinge linked to the C _H2_-C _H3_ domains from IgG1 or IgG4 ^44, 45^ or spacer domains from CD4 or CD8 ^46^. Reports have shown that CAR T cells containing a hinge from IgG1 Fc regions can bind to Fcγ receptors (FcγRs) and activate cells of the innate immune system ^44, 47^. Differences in potencies of individual CARs have been associated with choice of co-stimulatory domain used in CAR design, spacer length and its modification if any, target epitope density and location, and inter-membrane distance (for example, ^44, 45, 47– 51^). A study that used the γ chain of FcεRI receptor as a signaling domain in a CD30-specific CAR also showed that location of the epitope influenced activity, in that a CAR with a spacer region (C _H2_-C _H3_ domains) was less efficient in mediating T-cell activation than the same CAR without the spacer ^52^.

**Figure 1.**
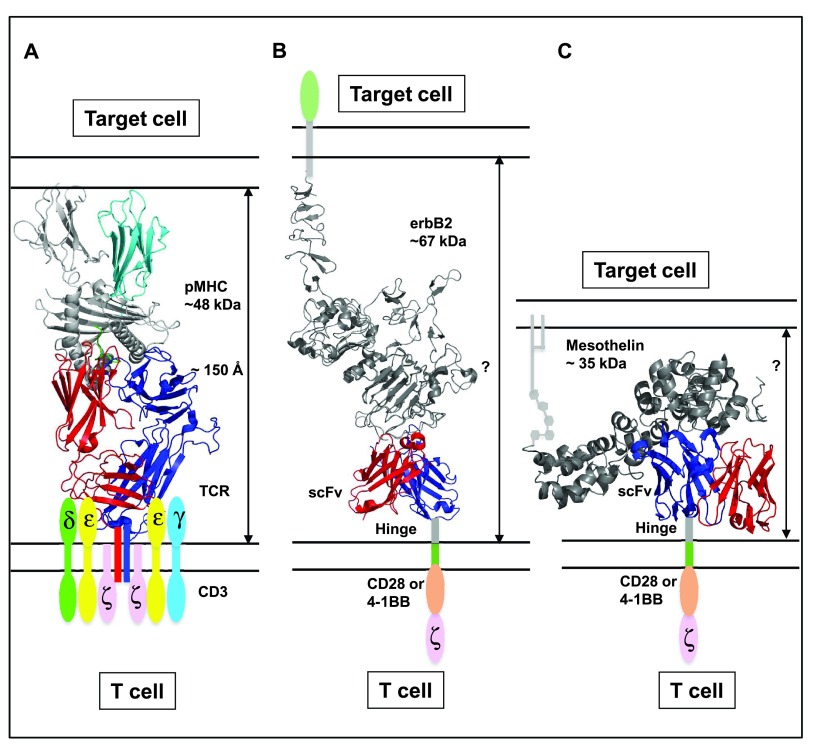
Dimensions of the interaction interfaces involving conventional αβ T-cell receptor (TCR) T cells and chimeric antigen receptor (CAR) T cells. ( **A**) TCRs on the surface of T cells interact with peptide-major histocompatibility complex (pMHC) complexes on the surface of target cells (antigen-presenting cells). This conserved interaction spans approximately 150 Å of inter-membrane space between the two cell types. TCRs assemble in the membrane of T cells with subunits of CD3 molecules (δ, ε, γ, and ζ) and CD4 or CD8 (not shown). Proximal, intracellular molecules initiate phosphorylation of CD3 subunits and subsequent signaling pathways. Structure of the Mel5 TCR in complex with MART-1 peptide bound to HLA-A2 is shown (PDB: 3HG1) ^116^. ( **B**, **C**) CARs typically contain single-chain variable fragment (scFv) domains (V _H_ and V _L_) of an antibody, linked to a hinge or spacer domain, transmembrane domain, and intracellular signaling domains (for example, co-stimulatory domains CD28 or 4-1BB and CD3ζ). CAR interacts with its antigen present on the target cell surface. Owing to potential differences in the size of the antigen and location of the epitope, the interaction interface of CAR-target antigen can be variable. In ( **B**), a representation of a CAR-target antigen interaction interface is shown by aligning the structures of an extracellular domain of the CAR target, ErbB2, in complex with the scFv of an anti-ErbB2 antibody, chA21 (PDB: 3H3B) ^63^, with the complete extracellular domain of ErbB2 (PDB: 1N8Z) ^64^. To illustrate the range of possible CAR interactions, in ( **C**) a representation of another CAR-target antigen interaction interface is shown for mesothelin, a membrane glycoprotein present on the cell surface of various cancers, including mesothelioma. Mesothelin was modeled by using the online tool “Phyre2” ^117^, followed by alignment with the domain of mesothelin that was crystallized with the Fab fragment of the anti-mesothelin monoclonal antibody MORAb-009 ^65, 118^. Note that although these are depicted as static structures, both protein dynamics and membrane mobility will also impact interface interactions.

Given the differences in extracellular domains and their antigen structures, the dimensions of the conserved TCR:pepMHC interface can differ substantially from a CAR:antigen interface. TCR:pepMHC interaction interfaces have been studied extensively ^1, 53– 56^ and crystal structures predict that the static inter-membrane distance for a TCR:pepMHC interaction is about 150 Å ( Figure 1A). This has been supported by the measurement of inter-membrane distances at TCR:pepMHC interfaces by electron microscopy ^57^. The issue of dimensions is important because the kinetic segregation model of TCR triggering proposes that the TCR:pepMHC distance has been optimized to include TCR:pepMHC and accessory receptor-ligand combinations at the synapse while excluding larger inhibitory tyrosine phosphatases (CD45 and CD148) ^57– 60^. Accordingly, TCR-mediated adoptive T-cell approaches will automatically retain the same evolutionarily optimized dimensions at the interface.

In contrast, distances at the CAR-target antigen interface will be highly variable depending on the size and epitope location of the target antigen. Figures 1B and 1C show a representation of two targets, ErbB2 and mesothelin, which are overexpressed in certain forms of cancer and have been used as targets for immune-based therapies, including CARs ^33, 61, 62^. Domains of ErbB2 and mesothelin have been crystallized in complex with antibody domains, allowing a schematic comparison of the possible interfaces that might exist when these proteins are targeted by CARs ^63– 65^. The ErbB2/scFv (antibody chA21) predicts that the extracellular domain of ErbB2 is about 115 Å in length, and the Ig-domains of the scFv measure about 35 to 40 Å. Thus, the static view of this complex indicates an interaction interface of about 150 Å, similar to the TCR:pepMHC interaction. In contrast, mesothelin has a very different domain structure with potentially shorter distances at the interaction interface. Both the ErbB2 and mesothelin models highlight the view that the location of the scFv epitope and the flexibility of the membrane-bound target antigen will have a direct influence on the dimensions of the interface. Of course, other factors such as the level of expression of the antigen, its inherent membrane mobility, and the affinity of the scFv will all influence the sensitivity of the CAR reactions. For example, the importance of epitope location has been emphasized by Abken and colleagues, who showed that transfer of a membrane-distal epitope to a membrane-proximal location resulted in improved CAR T-cell activation ^51^.

Determining the dimensions of CAR-target antigen interfaces is difficult, as often the structures of the extracellular regions of antigens are not known and, even if they are, the flexibility and membrane mobility of these structures cannot be predicted. Nevertheless, studies have indicated that altering the hinge/spacer domain of CAR constructs impacts their potency, supporting the idea that an optimal distance of interaction for CARs exists, as seen with the TCR:pepMHC interface ^45, 49, 50^. For example, ROR1-directed CAR constructs were effective with a shortened spacer domain for recognition and killing of ROR1-positive tumors because of the membrane-distal location of the ROR1 epitope ^48^. It was hypothesized that shortening the spacer may reduce the distance between T cell and target cell, hence allowing exclusion of inhibitory phosphatases (CD45) ^41^. Accordingly, a membrane-proximal epitope of ROR1 was efficiently recognized by CARs with a longer spacer ^45^. Similar results have been observed with CD19-directed CARs ^45^. As the size of the CAR:antigen dimension was increased with a CD22-directed CAR, the efficiency of target cell lysis was reduced, perhaps because the larger membrane interface permitted CD45-mediated dephosphorylation of substrates involved in T-cell signaling ^49^. These studies support the importance of maintaining an optimal inter-membrane distance between CAR and target antigen. They suggest that spacer lengths will need to be designed for individual CARs and that scFv fragments against a particular epitope of an antigen may need to be generated.

## Target antigen density and affinity requirements for TCRs and CARs

**Figure 2.**
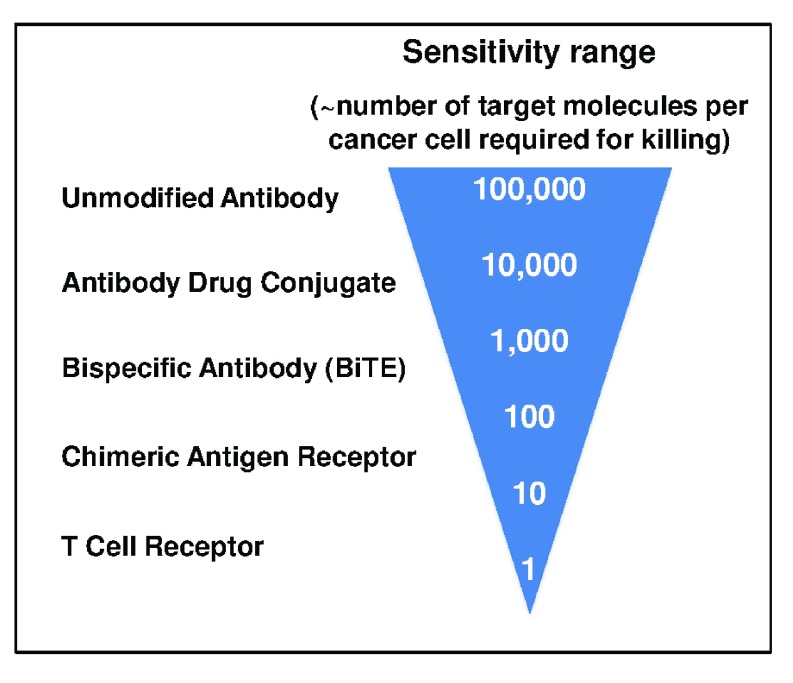
Sensitivity thresholds for various antibody or T-cell-based therapy modalities. A comparison of approximated sensitivity thresholds (that is, number of target molecules per cancer cell that are required for killing) that have been identified for antibody or T-cell-based approaches is depicted. Note that these are estimates and that within each category the sensitivity can be further influenced by various parameters, including the affinity of the receptor toward the target antigen. BiTE, bispecific T-cell engager.

T-cell triggering is not only sensitive to the distance between the antigen-receptor machinery and target cell antigen but also, of course, affected by the density of antigens presented to T cells bearing either the native TCR or the synthetic CAR ( Figure 2). It has been suggested that one pepMHC molecule may be sufficient to trigger T-cell activation ^6– 9^. A factor contributing to this sensitivity, suggested by the serial triggering hypothesis, is that the low affinity (fast off-rate) of the TCR:pepMHC interaction allows serial binding of multiple TCR/CD3 complexes by a single pepMHC complex, thereby amplifying the reaction ^66^. The extraordinary sensitivity of T cells is also explained by the action of co-receptors CD4 or CD8 which interact with invariant regions of class II or class I MHC and consequently bring the intracellular kinase Lck (lymphocyte specific receptor kinase) into proximity with the TCR/CD3 complex ^67^. Accordingly, TCR:pepMHC binding drives local aggregation of multiple TCR-pepMHC complexes, leading to Lck-mediated downstream signaling. In the absence of co-receptor, the sensitivity is reduced to about 30 or more pepMHCs, and this co-receptor-independent reaction requires a 10- to 100-fold higher affinity TCR:pepMHC interaction ^3, 8, 9, 68^.

Whereas T cells have evolved the machinery to optimize proximal signaling, synthetic CARs have adopted some but not all of these features. In fact, it could be argued that the antigens currently targeted by CARs dictate that the CARs have at least some properties that are different from TCRs. For example, the densities of cell surface antigens recognized by current-generation CARs are orders of magnitude higher than the densities of most specific pepMHC antigens, and these levels vary considerably from one target to another. CAR-based therapies have targeted antigens such as CD19, CD20, and ErbB2 that are expressed at densities that range from 10,000 to 1,000,000 molecules per tumor cell ^36, 62^. Depending on the system, there may be less information about the minimum number of antigen molecules required by CAR T cells to generate a response. With a unique glycopeptide-directed CAR system, the antigen density required for target cell lysis ranged from 300 to 3,000 epitopes per target cell, and this sensitivity was greater for the scFv used as a CAR compared with its use as a bispecific antibody (also known as BiTEs or bispecific T-cell engagers) ^36^. More recently, Watanabe and colleagues used a CD20-directed CAR system to establish that the threshold of antigen density required for target cell lysis was about 200 molecules per cell ^69^. On the other hand, a WT1-human leukocyte antigen (HLA)-A2-directed BiTE system has been shown to induce T-cell response and target-cell killing at 500 to 6,000 epitopes per cell ^70, 71^, whereas current antibody drug conjugates (ADCs) target antigens at significantly higher density (~10 ^4^ to ~10 ^5^) ^72^. It is important to note that, whether an antigen is targeted by a CAR, BiTE, or ADC product, each strategy can be optimized within its class to achieve recognition of the lowest antigen levels possible. Ultimately, this depends on the mechanism of action of their respective effector functions. With this in mind, TCRs expressed in T cells are currently the only mediator of activity endowed with the extreme sensitivity to recognize as few as one target molecule per tumor cell ( Figure 2).

Mechanistically, it is not clear whether CARs require the same extent of clustering as TCRs in the T-cell membrane and whether the signaling domains of CARs are actually generating the same stoichiometry and quantitative signaling amplification. The structural organization of CARs allows MHC-independent recognition of antigens, and hence there is no known contribution of co-receptors for further improvements in the sensitivity of this interaction. Accordingly, for each CAR system, it will be important to determine the range of target antigen densities toward which responses against tumor (but not normal tissue) are achieved. This might also require comparison of multiple scFv and CAR formats.

Naturally occurring TCRs possess low affinity toward their pepMHC ligands (dissociation constant [K _D_] ranging from 10 to 100 μM for foreign pepMHC) but still efficiently induce T-cell activation because of the contribution from co-receptors, serial triggering, and the stoichiometry of the CD3 complex. However, the affinity can be significantly lower for a self-cancer antigen (K _D_ of as low as about 1,000 μM) ^73^. Efforts to engineer TCRs for higher affinity are focused on two goals: first, to optimize the activity of CD8 T cells against self-pepMHC (class I) since the higher-affinity TCRs have been deleted during thymic selection ^73^ and, second, to redirect the activity of CD4 T helper cells against a class I MHC target since this interaction would not be able to use the cognate co-receptor CD8 ^74, 75^. Various studies have shown that there is an affinity threshold for TCRs in CD8 T cells (about 10 μM) and CD4 T cells (about 1 μM) beyond which they risk mediating self-peptide cross-reactivity ^76– 82^. Such affinity-enhanced TCRs (for example, against HLA-A1 restricted MAGE-A3 epitope) have been shown to cause off-target cross-reactivity resulting in lethality ^16, 83, 84^. It is generally accepted that there is no need to engineer TCR affinities below these “optimal” K _D_ values, but again each TCR requires individual testing to assess safety issues.

In contrast to TCRs, CARs typically contain scFv fragments from higher-affinity, monoclonal antibodies (K _D_ values in the range of 1 to 100 nM). It should be possible to select scFv affinities and CAR formats that avoid stimulation by normal tissues caused by on-target, off-tumor reactions, as apparently was observed with an ErbB2-directed CAR that was reactive with ErbB2 levels in the lungs of a patient ^62^. A recent study demonstrated the usefulness of low-affinity CAR T cells for distinguishing between malignant and normal cells (high antigen density versus low antigen density), but the high-affinity CAR T cells demonstrated a response that was independent of antigen density, indicating that, as with TCRs, understanding the affinity threshold window is important for controlling responses mediated by CARs ^85^.

To directly compare TCRs and CARs, our lab used the recognition domains (Vα and Vβ) of a high-affinity TCR, formatted either as a conventional full-length αβ TCR or as a CAR. Although both formats exhibited the same high affinity (30 nM), the TCR format was significantly more sensitive to pepMHC than the CAR format ^86^, showing that the native TCR/CD3 architecture has evolved more sensitivity than the current CAR designs. In another study, a TCR with a K _D_ value of about 1 μM was compared with CAR constructs (scFv) with K _D_ values of 30 or 400 nM against the same pepMHC ^87^. The lower-affinity TCR exhibited more potent cytotoxic activity and a high degree of specificity, whereas the high-affinity CAR exhibited reduced cytotoxic activity and loss of specificity.

There are various possible explanations for reduced sensitivity of CARs compared with conventional TCRs. The TCR/CD3 assembly in the membrane provides a total of 10 immunoreceptor tyrosine-rich activation motifs (ITAMs) that can be phosphorylated during TCR-pepMHC binding, initiating downstream signaling for T-cell activation ^42^. Current-generation CARs, on the other hand, contain fewer ITAMs (for example, three) which may yield a reduced level or kinetics (or both) of proximal signaling and corresponding lower sensitivities than TCRs, even when the TCR and CAR have identical binding affinities ^86^. The co-receptors CD4 or CD8 also participate in the binding and proximal signaling upon TCR interaction with pepMHC, whereas CAR-target antigen interactions do not appear to involve CD4 or CD8. Finally, normal TCR:pepMHC interactions can involve co-stimulatory receptors like CD28 and 4-1BB, further promoting full T-cell activation. In summary, although CAR design has included domains from important T-cell signaling domains (CD3ζ, CD28, or 4-1BB or a combination of these), the ability of CARs to multimerize in the membrane and to associate with other proximal signaling molecules is likely not as efficient as has evolved with TCR-associated machinery.

## Neoantigens as targets for TCR-mediated adoptive T-cell therapies

To date, efforts to treat cancer using TCR gene transfer into adoptive T cells have targeted shared cancer-associated antigens, including cancer/testis antigens ^10, 12, 14^. Whereas TCR-mediated targeting of NY-ESO has shown significant clinical promise ^12, 14, 15^, targeting of MAGE-A3 by two different TCRs led to lethal off-target reactions ^16, 83, 84^, and targeting of MART1 has been associated with significant on-target, off-tumor side effects ^11, 88^. Hence, there has been increasing interest in redirecting the activity of T cells against antigens that are unique to tumors in the form of mutated peptides known as neoantigens ^89– 91^. Although these antigens represent potential targets with a high level of antigen specificity, they are also typically unique to individual patients and thus pose significant challenges in the development of such personalized TCR-based therapeutics ^92^. Also, not all patients will possess T cells that mount a response to neoantigens, and one of the challenges will be to determine which neoantigen(s) are expressed at adequate levels on tumors to serve as targets for T-cell therapies.

Neoantigens arise as a consequence of somatic mutations in tumors and hence T cells against them are not subject to the same thymic tolerance mechanisms as self-antigens ^90, 93^. In fact, it is this property that allows cancer patients treated with checkpoint inhibitors against CTLA-4 and PD-1 to elicit immune responses against such neoantigens ^94– 96^. The identification of neoantigens from a patient’s tumor has been performed by whole exome sequencing, when RNA is available for RNASeq analysis (for example, ^97, 98^), followed by *in silico* screening for mutated peptides using HLA-binding and -processing prediction algorithms ^99– 106^. Candidate neoantigens are assessed for their ability to elicit T cells by use of synthetic peptides and autologous APCs ^107^, single-cell analysis using pep-HLA multimers ^108^, or expression of peptide cassettes in autologous APCs ^109^.

Although some approaches will use neoantigens in vaccine formulations, the use of neoantigens as targets in therapeutic numbers of T cells may benefit from the isolation of TCR genes from T cells that respond to the specific neoantigens, as has been done recently in a mouse system ^110^. Success here will depend on multiple factors. It has been shown that even with a significant number of predicted neoantigen epitopes, neoantigen-reactive T cells may be limited in some patients with cancer ^91, 111^. In a recent study, T cells isolated from healthy individuals were used to raise specific T cells against tumor neoantigens derived from patients ^91^. These results and others suggest that it will be possible to identify TCRs against specific neoantigens and to eventually use them to increase the number of therapeutic T cells by TCR gene transfer.

Neoantigens identified by tumor sequencing and bioinformatic analysis of MHC-binding (and possibly antigen-processing) algorithms are not all equal in terms of theoretical efficacy. It is useful to consider the classes that each neoantigenic peptide may represent. First, some predicted peptide epitopes will not be processed, or presented, at levels adequate to elicit T-cell immune responses. The magnitude of this class of neoantigen will vary depending on the robustness of the prediction algorithms for each HLA allele ^112, 113^.

**Figure 3.**
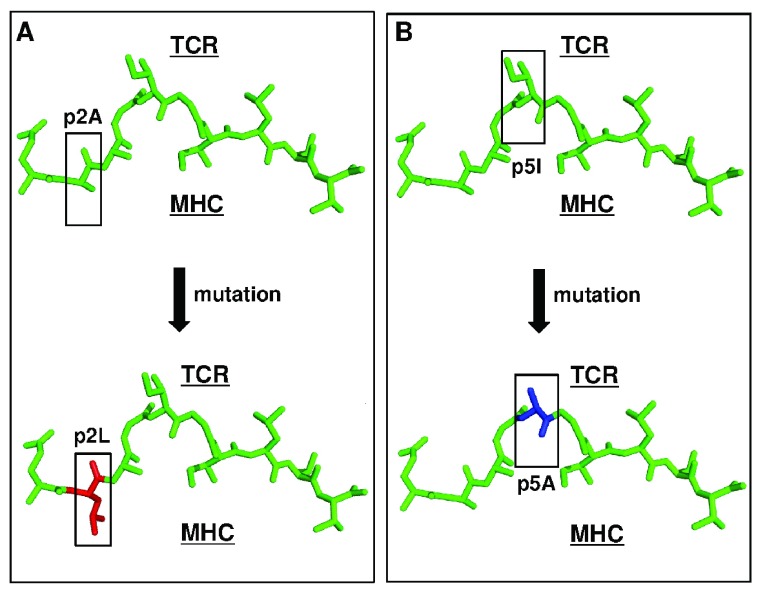
Neoantigens as targets for T cells: possible effects of single mutations. ( **A**) A mutation in a major histocompatibility complex (MHC) anchor residue (Ala to Leu; shown in red) is shown. Such a mutation could improve the binding of the peptide to MHC and thereby increase the number of peptide-MHC (pepMHC) complexes on a target cell (antigen-presenting cell). ( **B**) A mutation (Ile to Ala; shown in blue) in a residue that points away from the MHC but is in a position to interact with a T-cell receptor (TCR) is shown. Since the normal repertoire of peripheral T cells has not been tolerized against the mutated peptide, there are likely to be some TCRs that have binding affinities for this pepMHC complex that drive T-cell activity. Alternatively, a combination of effects shown in ( **A**) and ( **B**) might be achieved when the mutated residue impacts affinity for the MHC but also alters the conformation of the exposed peptide which could interact with a TCR. For reference, the MART-1 peptide is shown (PDB: 4QOK) and the specific mutations were either present in a known structure (PDB: 3HG1) or modeled by using PyMol.

A second class of neoantigens will be those peptides that have been identified because they were predicted to have greater binding, than the wild-type peptide, to an HLA allele (for example, peptides with a mutation in a known anchor residue or other residues that point toward MHC) ( Figure 3A). Such a mutation may increase binding of the peptide to the MHC molecule and hence will impact the number of the neoantigen/HLA complexes on the tumor cell surface (that is, density) compared with the number of the wild-type antigen/HLA complexes. Mechanistically, this outcome (higher pepMHC surface levels) is similar to upregulated cancer-associated self-peptides if one assumes that the mutation does not impact the conformation of the peptide region presented to the T cell. T cells with TCRs against these neoantigens, like TCRs against self-peptide cancer-associated antigens, will in general be of lower affinity as T cells expressing higher-affinity TCRs will have been deleted during thymic selection ^73^.

A third class of neoantigens consists of those peptides that contain a mutation in a residue that points toward the TCR and hence could impact binding to TCR ( Figure 3B). In principle, these mutated peptides could serve as optimal targets since they will be more immunogenic; that is, peripheral T cells will perceive these peptides as non-self/foreign since the T cells have not been subjected to thymic negative selection.

A fourth class of neoantigens includes peptides that have a mutation in a residue that impacts the interaction both with the TCR and with the MHC. These neoantigens could potentially have the strongest impact since the number of neoantigen/HLA complexes would be higher than the wild-type peptide/HLA (assuming the mutation increased binding to the HLA) and the neoantigen-peptide surface recognized by the TCR would differ from the surface of the wild-type peptide, such that T cells with TCRs exhibiting higher affinity would be available in the periphery. We have shown that the number of positions in a peptide that could impact both MHC and TCR binding varies among different MHC alleles ^114^. It will be important to examine these issues with single amino acid peptide variants tested in many different HLA alleles. Such detailed analysis of mutations at each residue in peptide antigens should provide a better understanding of the potential potency of neoantigens and help guide the selection of neoantigens for adoptive T-cell therapies. Although we have focused here on neoantigens that exhibit single-site mutations, there is the potential for other classes of neoantigens that derive from more extensive mutation, including insertions, deletions, or even glycosylation aberrancies ^115^.

## Concluding remarks

Recent efforts to engineer T cells against cancer have taken two different approaches. Conventional TCR-mediated therapies take advantage of the well-known specificity and sensitivity of normal T-cell activities. Studies have begun to explore the possibilities of engineering T cells by using TCRs against a patient’s neoantigens. Many of these represent intracellular antigens that would not be accessible by conventional antibody (or CAR) therapies. The selection of the most efficacious neoantigen(s) as targets should consider the mechanistic impact of the mutation and the selection algorithms used to identify these potential antigens.

CAR-mediated approaches have tremendous potential against tumor cell surface antigens but their mechanism of action is less fixed than the TCR because of features that vary among different target antigens and CAR constructs. Thus, it is important to find an appropriate balance between antigen on the one side (antigen surface level, epitope location, and antigen mobility) and CAR structure (CAR surface level, affinity, and signaling domains) on the other.

## Abbreviations

ADC, antibody drug conjugate; APC, antigen-presenting cell; BiTE, bispecific T-cell engager; CAR, chimeric antigen receptor; HLA, human leukocyte antigen (refers to human major histocompatibility complex alleles); ITAM, immunoreceptor tyrosine-rich activation motif; K _D_, dissociation constant; Lck, lymphocyte-specific receptor kinase; MHC, major histocompatibility complex; pepMHC, peptide complexed with major histocompatibility complex; scFv, single-chain fragments variable; TCR, T-cell receptor.
